# A Case Report of Ropeginterferon Alfa-2b for Polycythemia Vera during Pregnancy

**DOI:** 10.3390/hematolrep15010018

**Published:** 2023-03-02

**Authors:** Su-Yeon Bang, Sung-Eun Lee

**Affiliations:** Department of Hematology, Seoul St. Mary’s Hospital, College of Medicine, The Catholic University of Korea, Seoul 06591, Republic of Korea

**Keywords:** case report, myeloproliferative neoplasms, polycythemia vera, pregnancy, ropeginterferon alfa-2b, interferon, South Korea

## Abstract

Myeloproliferative neoplasms (MPN) such as essential thrombocythemia (ET) and polycythemia vera (PV) are rare during pregnancy. However, they are harmful because they are associated with an increased risk of thromboembolic, hemorrhagic, or microcirculatory disturbances or placental dysfunction leading to fetal growth restriction or loss. Low-dose aspirin and low-molecular-weight heparin (LMWH) are recommended to reduce pregnancy complications, and interferon (IFN) is the only treatment option for cytoreductive therapy based on the likelihood of live birth in pregnant women with MPN. Since ropeginterferon alfa-2b is the only available IFN in South Korea, we present a case report of ropeginterferon alfa-2b use during pregnancy in an MPN patient. A 40-year-old woman who had been diagnosed with low-risk PV in 2017 and had been maintained on phlebotomy, hydroxyurea (HU), and anagrelide (ANA) for 4 years was confirmed as 5 weeks pregnant on 9 December 2021. After stopping treatment with HU and ANA, the patient showed a rapid increase in platelet count (1113 × 10^9^/L to 2074 × 10^9^/L, normal range, 150–450 × 10^9^/L) and white blood cell count (21.93 × 10^9^/L to 35.55 × 10^9^/L, normal range, 4.0–10.0 × 10^9^/L). Considering the high risk of complications, aggressive cytoreductive treatment was required, for which we chose ropeginterferon alfa-2b, as it is the only available IFN agent in South Korea. The patient underwent 8 cycles of ropeginterferon alfa-2b over 6 months during pregnancy and delivered without any neonatal or maternal complications. This case report highlights the importance of considering treatment options for MPN patients who are pregnant or planning a pregnancy, as well as the need for further investigation into the safety and efficacy of ropeginterferon alfa-2b in this population.

## 1. Introduction

The 2016 World Health Organization (WHO) classification system recognizes myeloproliferative neoplasms (MPN) as one of several myeloid malignancies [1,2]. In routine clinical practice, MPN refers to the three *JAK2/CALR/MPL* mutated entities, namely, polycythemia vera (PV), essential thrombocythemia (ET), and primary myelofibrosis (PMF); these disorders are characterized by stem cell-derived clonal myeloproliferation with mutually exclusive *JAK2*, *CALR*, and *MPL* mutations [3,4]. Although they are a heterogeneous group of disorders, MPN share common complications, such as thrombotic and hemorrhagic events; debilitating microvascular, constitutional, or other disease-related symptoms; and fibrotic or leukemic transformation [5]. Among them, PV is characterized by mutations in exon 14 or exon 12 of *JAK2* and is phenotypically associated with one or more of erythrocytosis, systemic symptoms (for example, headache, red skin, tiredness, dizziness, etc.), major thrombosis, and microvascular symptoms [6].

In 2018, the European LeukemiaNet (ELN) provided consensus recommendations for the management of chronic myeloproliferative neoplasms [7], but there are still many unmet needs for the satisfactory management of patients with these conditions. For many decades, hydroxyurea (HU) has been a mainstay of therapy; however, the potential therapeutic options for PV have now expanded beyond HU, with the approval of ropeginterferon alfa-2b and the *JAK1/JAK2* inhibitor ruxolitinib [8]. 

Among cytoreductive agents used to treat PV, interferon (IFN) alfa has shown efficacy for more than 30 years. The mechanisms of the action of IFN alfa have been ascribed to its anti-proliferative, pro-apoptotic, anti-angiogenic, and immunomodulatory effects [9,10,11]. Additionally, it has been consistently reported to have a disease-modifying capacity by selectively decreasing the malignant stem cell pool and inducing durable molecular remissions in some patients [12,13,14,15]. In ELN 2021 recommendations for the management of PV, the expert panel recommended either pegylated or non-pegylated IFN alfa as cytoreductive drug therapy in patients younger than 60 years with PV, especially because their potentially longer lifespan might be associated with a higher HU cumulative incidence confirmed to be of secondary malignancies and myelofibrosis [8].

IFN alfa has been used during pregnancy because it does not significantly increase the risk of major malformation, miscarriage, stillbirth, or preterm delivery above general population rates [16]. Moreover, higher live birth rates associated with the use of IFN were observed in a systematic review of MPN pregnancies and suggest a potential reduction in disorders associated with placental dysfunction [5]. However, the clinical use of IFN alfa has mainly been limited because of poor tolerance among patients exposed to daily injections of the compound. To improve tolerability, pegylated forms of IFN alfa have been developed that can be administered with less frequent dosing [17]. Ropeginterferon alfa-2b, a next-generation, mono-pegylated IFN alfa-2b isoform, is developed for treating myeloproliferative neoplasms [18]. This drug is the only IFN alfa formulation currently approved in Europe and the USA for patients with PV [8].

The present study describes the case of a 40-year-old pregnant woman with *JAK2V617F* mutation diagnosed as PV according to the WHO criteria [1]. This was the first use of ropeginterferon alfa-2b during pregnancy in South Korea.

## 2. Case Presentation

A 36-year-old woman was hospitalized for further evaluation after her red blood cell (RBC) and white blood cell (WBC) counts were found to be elevated during a routine medical checkup in December 2017. Her WBC was 13.87 × 10^9^/L (normal range, 4.0–10.0 × 10^9^/L) with hemoglobin (Hb) of 18.2 g/dL (normal range, 12.0–16.0 g/dL), hematocrit (Hct) 55.6% (normal range, 34.0–49.0%), and platelet (PLT) of 821 × 10^9^/L (normal range, 150–450 × 10^9^/L). She had no underlying diseases and reported only mild fatigue at the time of her evaluation, with no other significant symptoms. She was positive for the *JAK2V617F* (homozygous, variant allelic frequency 63.96%) and had no history of thrombotic events. She was diagnosed with low-risk PV in 2017, according to WHO criteria [1,19]. She started treatment with low-dose aspirin and phlebotomy. Because of progressive and persistent leukocytosis and extreme thrombocytosis, she maintained treatment with phlebotomy, HU, and anagrelide (ANA) for 4 years and underwent frequent dose adjustments to 500 mg or 1000 mg/d for HU and 1.0 or 1.5 mg/d for ANA based on complete blood count (CBC) results and tolerability of treatment. 

On 9 December 2021 at 40 years, she was confirmed to be 5 weeks pregnant, and all cytoreductive treatments were discontinued except for low-dose aspirin, due to its known teratogenic risk. She was aware of the pregnancy risks and decided to proceed with the pregnancy as it was their first pregnancy. On 17 December 2021, her WBC was 21.93 × 10^9^/L (absolute neutrophil count (ANC) 17.98 × 10^9^/L) with Hb 13.8 g/dL, Hct 44.8%, and PLT of 1113 × 10^9^/L. Two weeks after stopping treatment with HU and ANA, CBC values increased rapidly as follows: WBC 35.55 × 10^9^/L (ANC 30.22 × 10^9^/L), Hb 14.2 g/dL, Hct 47.4%, and PLT 2074 × 10^9^/L. The therapeutic options for cytoreduction in pregnant patients were highly restricted. In addition, no cases of IFN treatment have been reported in pregnant patients with PV in South Korea. She expressed significant concerns regarding the potential adverse effects on the fetus. After careful consideration, she opted to initiate ropeginterferon alfa-2b therapy. However, at the time of treatment, the medication was not readily available in South Korea, necessitating the process of submitting applications and importing the drug. This resulted in a delay of approximately 6 weeks in administration.

**Figure 1:**
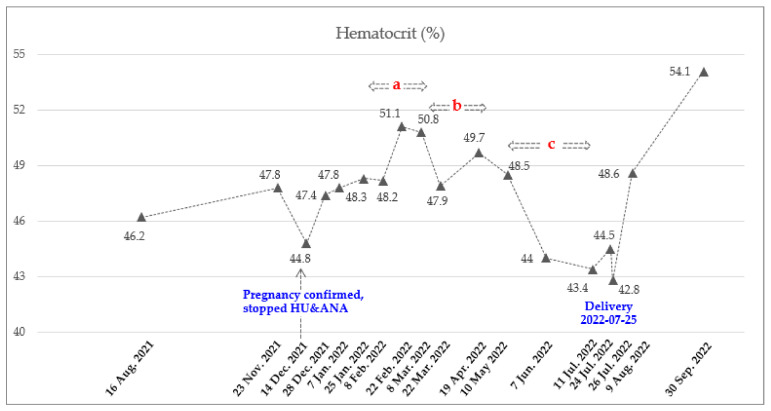
Dynamics of Hct count during pregnancy. Total of 8 doses of Ropeginterferon alfa-2b from 8 February 2022 to 11 July 2022. Injection schedule and dose: A. Every 2 weeks (250-350-500 mcg), b. Every 3 weeks (500 mcg), and c. Every 4 weeks (500 mcg).

**Figure 2:**
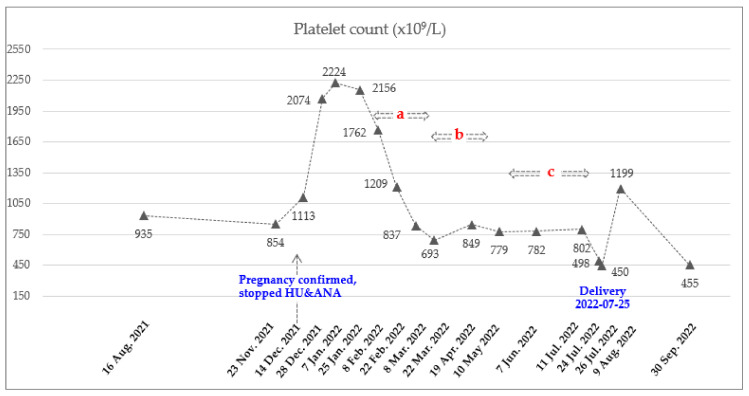
Dynamics of PLT count during pregnancy. Total of 8 doses of Ropeginterferon alfa-2b from 8 February 2022 to 11 July 2022. Injection schedule and dose: A. Every 2 weeks (250-350-500 mcg), b. Every 3 weeks (500 mcg), and c. Every 4 weeks (500 mcg).

The recommended starting dose for ropeginterferon alfa-2b is 100 mcg with a gradual increase of 50 mcg every two weeks [18]. However, due to the rapid rise in WBC and PLT counts, a starting dose of 250 mcg was administered subcutaneously on 8 February 2022, based on clinical trial experiences (clinicaltrial.gov: NCT04285086, ICTRP no.: KCT0006138) and published real-world data [20]. To achieve a rapid hematological response, it was planned to increase the dose rapidly. The most commonly reported adverse events in the ropeginterferon alfa-2b were thrombocytopenia, leukopenia, anemia, increased γ-glutamyltransferase, fatigue, increased alanine aminotransferase (ALT) and aspartate aminotransferase (AST), headache, arthralgia, and dizziness [18]. She visited the hospital every two to three weeks for blood testing, which included CBC and liver function tests. During these visits, she also asked the patient about their subjective symptoms. Two weeks after receiving the first dose, the patient was found to be in good condition based on a blood test, and the ropeginterferon-alfa 2b dose was increased to 350 mcg. This dose was increased to 500 mcg to achieve a rapid hematologic response. Two weeks later, the AST and ALT levels were slightly elevated, at 59 (normal range, 0–32 U/L) and 80 (normal range, 0–33 U/L) U/L, respectively. As a result, the administration of ropeginterferon alfa-2b was temporarily suspended. A week later, she was in good condition and resumed medication. Considering the observed trend of decreasing CBC after treatment (Figure 1 and Figure 2), it was decided to administer the medication every three weeks. Since then, all her liver function levels have remained in the normal range. The maintained dose was 500 mcg, and the frequency of injection was reduced from every 3 weeks and then every 4 weeks considering the adequate response. She underwent 8 cycles of ropeginterferon alfa-2b during pregnancy, with a final date of injection of 11 July 2022. She stopped treatment with low-dose aspirin on 10 July 2022 and received LMWH from 11 to 23 July 2022 to prepare for delivery (Table 1). The baby was delivered by cesarean section on 25 July 2022, at 37 weeks 6 days of gestation (weight 3.13 kg, male) without any neonatal and maternal complications.

This case report was approved by the Institutional Review Board of Seoul St. Mary’s Hospital and was conducted according to the tenets of the Declaration of Helsinki. 

## 3. Discussion

MPN are mainly diagnosed between the ages of 50 and 70 years, and the incidence in young patients is very low [21,22]. In a nationwide study in South Korea, a higher incidence of MPN was seen in older age groups, and PV and MF were more frequent in men than in women [22]. Men show a large predominance of PV in young patients in South Korea (male-to-female ratio 8.13:1) [23]. However, with improvements in diagnostic ability and increasing access to mutational analysis, MPN continue to be diagnosed earlier [1,24,25]. Combined with the societal trend of increasing mean maternal age, MPN are increasingly being identified in women who are pregnant or planning a pregnancy [5]. The true incidence of pregnancy in MPN has not been ascertained; however, pregnancy events have been reported most frequently in ET, followed by PV, and rarely in PMF [26]. Both pregnancy and MPN increase the risk of thrombosis due to the hypercoagulable environment they create. 

Thrombosis is the primary cause of maternal death in normal pregnancies. Thrombotic occlusion of the placental circulation leading to infarction may be a result of placental dysfunction or may occur as an independent mechanism affecting pregnancy outcomes [26]. It is well known that MPN patients experience a higher rate of hemorrhagic events [27], which indicates an increased risk of hemorrhagic events leading to fetal loss in MPN patients during pregnancy. Moreover, placental infarction often can be responsible for intrauterine fetal growth retardation during pregnancy of MPN patients [28]. According to a systemic review study, the overall live birth rate was 71.3% [5]. 

Pregnancy is considered a high-risk clinical situation in patients with PV and ET. In previous studies, recommended treatments are (1) low-dose aspirin daily, (2) prophylactic LMWH throughout pregnancy and for 6 weeks postpartum, and (3) cytoreductive therapy with IFN or peginterferon alfa-2a [29,30,31,32]. However, there are not sufficient data on the use of peginterferon alfa-2a (risk category C) in pregnancy, and it should be considered carefully only if the benefits outweigh the potential risk to the fetus. 

There is no recommendation for MPN in pregnancy in the most recent ELN guideline [8], but aspirin, LMWH, and IFN alfa are recommended as treatment options in the previous version [33]. Especially, IFN alfa treatment is highly recommended for high-risk pregnancy in MPN defined as (1) previous major thrombosis or severe pregnancy complications and/or (2) PLT > 1500 × 10^9^/L, and/or (3) previous major bleeding event [32]. 

The Mayo Clinic’s latest guidelines suggest a more aggressive approach to treating pregnancy in patients with MPN. All high-risk ET and PV patients with a history of thrombosis should receive cytoreductive therapy in the form of IFN alfa. Additionally, low-risk PV patients with a history of poorly controlled hematocrit levels, prominent splenomegaly, or recurrent fetal loss may also benefit from IFN-alfa therapy. Systemic anticoagulation in the form of LMWH is advised in patients with a history of venous thrombosis [29]. 

Ropeginterferon alfa-2b is a next-generation IFN with a mono-pegylated recombinant analog and is manufactured by PharmaEssentia with an improved pegylated molecular coupling procedure [17]. Ropeginterferon alfa-2b, compared to conventional IFN alfa compounds, consists of a single positional isomer which leads to an extended elimination half-life. This results in less frequent dosing schedules, such as every other week or monthly during maintenance therapy, and improved tolerability, promoting long-term patient compliance [18]. Ropeginterferon alfa-2b has been approved in Europe and the USA for treatment of PV on the basis of phase 2 and 3 studies [17,18] and is available in the EU, USA, Taiwan, and South Korea. Since ropeginterferon alfa-2b is a newly developed IFN, there are no or limited data on the treatment of pregnant women with MPN. One case by the European Hematology Association (EHA) showed that ropeginterferon alfa-2b, at a dose of 350 mcg every other week, can be used safely in a pregnant patient with PV [34]. 

The case of PV in pregnancy presented herein was not only categorized as an elderly primigravida, which contributes to adverse pregnancy outcomes [35], but also had a high risk of complications due to uncontrolled PLT levels. In general, PLT counts in all women are expected to decrease by 17% throughout pregnancy because of dilution by the increased plasma volume and the increase in splenic and placental circulation [36]. It is also observed in pregnant women with ET. In a recent report, an average PLT count that had decreased by 43% was observed in 53 women with ET during pregnancy, with a nadir at delivery [30]. Different from ET pregnancy, the median PLT count at diagnosis was 656 (484–985) × 10^9^/L in 25 pregnancies with PV. This indicated an elevated PLT count compared with ET [37], but there are insufficient data for PLT trend analysis in PV pregnancy. A high PLT level was observed and increased rapidly in our case. Therefore, more aggressive cytoreductive therapy was needed. In addition to ANA (category C) and HU (category D), ruxolitinib (*JAK1/JAK2* inhibitor) was also classified as category C, and there was no data for pregnant women. So ruxolitinib could not be considered in our case. Since ropeginterferon alfa-2b is the only available IFN in South Korea and the efficacy and safety of the treatment of PV patients are known, we used ropeginterferon alfa-2b for 6 months during pregnancy. The patient not only showed an adequate hematologic response during the treatment but also delivered without any complications. The recommended ropeginterferon alfa-2b starting dose is 100 mcg and gradually increases by 50 mcg every two weeks [18]. Here, we applied a rapid dose increment considering treatment urgency based on the clinical experiences of ongoing trials (clinicaltrial.gov: NCT04285086, ICTRP no.: KCT0006138) and published real-world data [20]. We observed rapid dose increases and reduced frequency of injection schedule in this case, but no serious adverse reactions were observed during the treatment.

## 4. Conclusions

In conclusion, this case report suggests the possibility of safe use, but more cases need to be studied to confirm the safety of using ropeginterferon alfa-2b for cytoreductive therapy in pregnant patients with PV. Additionally, the optimal starting time, dose, and schedule of treatment also warrant further investigation.

## Figures and Tables

**Table 1:** Laboratory results and details of treatment status during pregnancy.

Date	Hct (%)	PLT (×10^9^/L)	Hb (g/dL)	WBC (×10^9^/L)	ANC (×10^9^/L)	Treatment Status
18 December 2017	55.6	821	18.2	13.87		At the time of diagnosis
December 2020~November 2021 (Mean ± SD)	46.3 ± 2.2	835 ± 130	13.9 ± 0.7	18.65 ± 0.58	13.88 ± 0.71	Phlebotomy and HU, ANA
14 December 2021	44.8	1113	13.8	21.93	17.98	Pregnancy confirmed, stopped HU and ANA
28 December 2021~25 January 2022 (Mean ± SD)	47.8 ± 0.5	2151 ± 75	14.8 ± 0.5	28.24 ± 6.73	23.05 ± 6.62	
8 February 2022	48.2	1762	14.9	22.06	18.53	Ropeginterferon alfa-2b 250 mcg
22 February 2022	51.1	1209	15.9	14.23	10.67	Ropeginterferon alfa-2b 350 mcg
8 March 2022	50.8	837	15.8	10.17	7.42	Ropeginterferon alfa-2b 500 mcg
22 March 2022	47.9	693	15.2	10.51	8.09	Ropeginterferon alfa-2b 500 mcg every 3 weeks (2 times)
19 April 2022	49.7	849	15.9	14.2	11.79	
10 May 2022 ~11 July 2022 (Mean ± SD)	45.3 ± 2.8	788 ± 13	14.7 ± 0.9	16.46 ± 2.83	13.54 ± 2.73	Ropeginterferon alfa 2b 500 mcg every 4 weeks (3 times) Final date of injection 11 July 2022
24 July 2022	44.5	498	14.3	15.93	12.82	LMWH (11 July 2022~23 July 2022)
26 July 2022	42.8	450	14.1	14.46	11.96	Delivery (25 July 2022)
9 August 2022	48.6	1199	15.9	21.53	18.09	Phlebotomy and HU, ANA
30 September 2022	54.1	455	17.2	15.68	12.54	

Hct: Hematocrit, PLT: Platelet, Hb: Hemoglobin, WBC: White Blood Cell Count, ANC: Absolute Neutrophil Count, HU: Hydroxyurea, ANA: Anagrelide, and LMWH: Low-molecular-weight heparin.

## Data Availability

The data presented in this study are available on request from the corresponding author.
